# Identification and Characterization of the Glutathione S-Transferase Gene Family in Blueberry (*Vaccinium corymbosum*) and Their Potential Roles in Anthocyanin Intracellular Transportation

**DOI:** 10.3390/plants13101316

**Published:** 2024-05-10

**Authors:** Xuxiang Wang, Jiajia Dong, Yiting Hu, Qiaoyu Huang, Xiaoying Lu, Yilin Huang, Mingyang Sheng, Lijun Cao, Buhuai Xu, Yongqiang Li, Yu Zong, Weidong Guo

**Affiliations:** 1 College of Life Sciences, Zhejiang Normal University, Jinhua 321004, China; xxwang@zjnu.edu.cn (X.W.); djj1134817956@163.com (J.D.); hyt1355951623@163.com (Y.H.); hqy_00@163.com (Q.H.); luxy0262@163.com (X.L.); huang_yl0023@163.com (Y.H.); sheng657@zjnu.edu.cn (M.S.); 13587217324@163.com (L.C.); flipped5723@163.com (B.X.); lyq@zjnu.cn (Y.L.); 2 Zhejiang Provincial Key Laboratory of Biotechnology on Specialty Economic Plants, Zhejiang Normal University, Jinhua 321004, China

**Keywords:** glutathione S-transferases, blueberry, expression patterns, anthocyanin intracellular transport

## Abstract

The glutathione S-transferases (GSTs, EC 2.5.1.18) constitute a versatile enzyme family with pivotal roles in plant stress responses and detoxification processes. Recent discoveries attributed the additional function of facilitating anthocyanin intracellular transportation in plants to GSTs. Our study identified 178 *VcGST* genes from 12 distinct subfamilies in the blueberry genome. An uneven distribution was observed among these genes across blueberry’s chromosomes. Members within the same subfamily displayed homogeneity in gene structure and conserved protein motifs, whereas marked divergence was noted among subfamilies. Functional annotations revealed that *VcGSTs* were significantly enriched in several gene ontology and KEGG pathway categories. Promoter regions of *VcGST* genes predominantly contain light-responsive, MYB-binding, and stress-responsive elements. The majority of *VcGST* genes are subject to purifying selection, with whole-genome duplication or segmental duplication serving as key processes that drive the expansion of the *VcGST* gene family. Notably, during the ripening of the blueberry fruit, 100 *VcGST* genes were highly expressed, and the expression patterns of 24 of these genes demonstrated a strong correlation with the dynamic content of fruit anthocyanins. Further analysis identified *VcGSTF8*, *VcGSTF20*, and *VcGSTF22* as prime candidates of *VcGST* genes involved in the anthocyanin intracellular transport. This study provides a reference for the exploration of anthocyanin intracellular transport mechanisms and paves the way for investigating the spectrum of GST functions in blueberries.

## 1. Introduction

Glutathione S-transferases (GSTs, EC 2.5.1.18) constitute an ancient and complex protein superfamily that is commonly distributed in plants, animals, fungi, and bacteria [1]. They play vital roles in various key metabolic processes in different creatures [2]. Glutathione S-transferases in plants are generally soluble proteins. They are primarily observed in dimers assembled from identical or similar subunits. The subunit assembly modes directly influence the diversity of GSTs in plants [3]. Additionally, monomeric and trimeric GSTs were discovered in some plants [4]. Each subunit of a typical glutathione transferase contains an N-terminal domain constituting the glutathione binding site (G-site) and a C-terminal domain composing the hydrophobic substrate binding site (H-site). The N-terminal domain exhibits higher conservation than the C-terminal domain [5]. GSTs showed highly conserved structural features but relatively poor sequence homology among members. Their classification is complex due to extreme sequence variances among different subfamilies. According to sequence similarity, catalytic residues, and evolutionary relationships, the GST superfamily can be divided into 14 classes: Tau (U), Phi (F), Theta (T), Zeta (Z), Lambda (L), Gamma subunit of the eukaryotic translation elongation factor 1B (EF1BG), dehydroascorbate reductase (DHAR), metaxin, tetrachlorohydroquinone dehalogenase (TCHQD), GSTs with two thioredoxins (GST2N), microsomal prostaglandin E synthase type 2 (mPGES2), hemerythrin, iota, and glutathionyl-hydroquinone reductases (GHR) [6,7]. Tau and Phi are the most general classes in the GST superfamily. Tau, Phi, Lambda, and TCHQD classes are considered to be plant-specific. As a multiple-gene and functional protein family, glutathione S-transferases are ubiquitously involved in regulatory and catalytic networks of plant development, playing indispensable roles in various metabolic activities [8]. Xenobiotics detoxification is one of the vital functions of GSTs. Their prototypical reaction pattern is that GSTs catalyze the conjugation of glutathione (GSH) with various cytotoxic substrates, thereby enhancing solubility, reducing toxicity, or transporting them to appropriate cellular compartments [9,10]. GSTs also play important roles in abiotic and biotic stresses [11], plant development [12], signal transduction [13], and secondary metabolism [14]. In recent years, the roles of GSTs in anthocyanin intracellular transport were extensively reported.

Anthocyanins are a class of phenolic secondary metabolites that are widely present in plants, mostly in glycosylated forms [15]. They are derived from anthocyanidins through modifications such as hydroxylation, methylation, glycosylation, and acylation. To date, over 30 anthocyanidins and more than 700 anthocyanins have been identified. As a class of water-soluble natural pigments, anthocyanins are the fundamental coloring substances in plant tissues and reproductive organs [16]. They also assume significant positions in plant propagation, physiology, ecology, and defense mechanisms [17]. In recent years, with the application of advanced chromatographic and spectroscopic techniques, numerous anthocyanins have been extracted from plant tissues [18]. They have been widely used in pharmaceutical, healthcare, food processing, and cosmetic fields, exhibiting tremendous health and economic values [19]. Anthocyanins are synthesized in the cytoplasm but eventually need to be transported to the vacuoles for storage. Although the biosynthetic pathways of anthocyanins are relatively well understood, the mechanisms of their intracellular transport are poorly known. Recently, three hypothetical mechanisms for anthocyanin intracellular transport to the vacuoles have been proposed, including GST-mediated transport, vesicular trafficking, and transporter protein-mediated transport [20]. In the GST-mediated pathway, GSTs act as noncatalytic carriers to bind anthocyanins and transport them from the cytoplasm to the vacuoles, ultimately leading to the accumulation of anthocyanins in the vacuoles. To date, anthocyanin intracellular transport GSTs proteins have been reported in many plants, such as TT19 in Arabidopsis [21], VvGST1 and VvGST4 in grape [22], LcGST4 in lychee [23], MdGSTF6 in apple [24], and PpGST1 in peach [25], suggesting that GSTs play pivotal roles in anthocyanin intracellular transportation.

Blueberries (*Vaccinium corymbosum*) have attracted a great deal of attention due to their unique flavor and abundant nutrients and bioactive compounds [26]. Anthocyanin flavonoids account for 60% of the total polyphenol content in mature blueberries, and as such, they have higher anthocyanin levels than most other fruits. Anthocyanins not only confer the distinctive coloration of blueberries, but also deliver tremendous health benefits to humans [27,28]. The synthesis, intracellular transport, and accumulation of anthocyanins involve various functional proteins. Increasing evidence indicates that GST proteins are closely associated with anthocyanin intracellular transport. Although the *GST* genes and their encoding anthocyanin intracellular transport proteins have been identified in some plants, relative studies in blueberries remain scarce. The main objectives of this study are three-fold: (1) comprehensively identifying and characterize the blueberry *GST* gene family, including the physicochemical properties, phylogenetic relationships, gene structural features, conserved protein motifs, cis-regulatory elements, functional enrichment, and gene duplication and evolution of their encoded proteins; (2) analyzing the expression patterns of *VcGST* genes during blueberry fruit development; (3) integrating the gene expression and anthocyanin content of blueberry fruits to predict potential GST proteins involved in anthocyanin intracellular transport. Through this study, we aim to lay a foundation for further elucidation of the functions of *VcGST* genes and for unraveling the molecular mechanisms underlying anthocyanin intracellular transport in blueberries.

## 2. Results

### 2.1. Identification and Characterization of GST Genes

**Figure 1:**
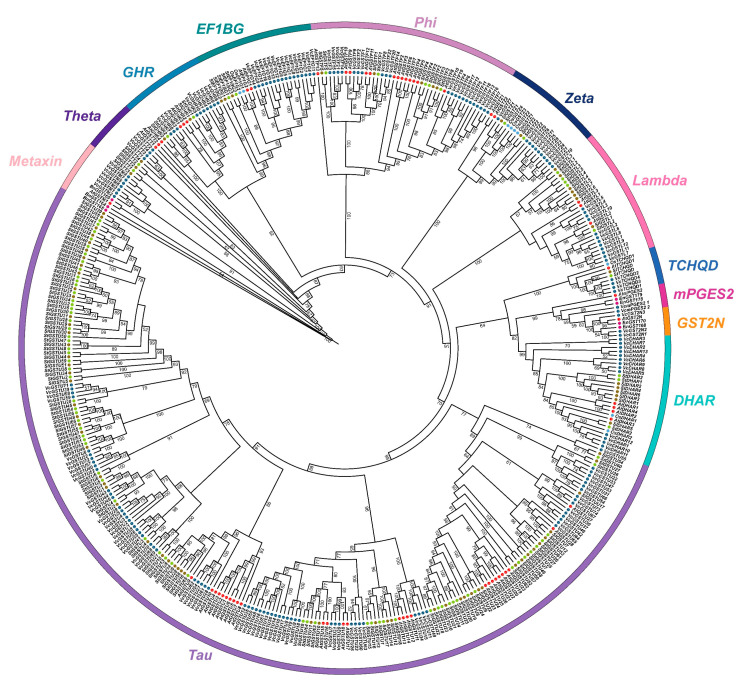
Phylogenetic tree of 430 GST proteins from *A. thaliana*, *O. sativa*, *B. napus*, *Solanum tuberosum*, *S. lycopersicum* and *V. corymbosum*. Genes from different species were marked with dots in different colors. Subfamilies were marked with colored strips. Digits on the branches represent bootstrap values based on 1000 iterations.

A total of 178 *GST* genes were identified in the blueberry genome. They were named based on their subfamilies and physical locations on chromosomes (Figure 1, Table S1). The lengths of GST proteins ranged from 165 amino acids (VcDHAR4, VcGSTU16, VcGSTU48) to 858 amino acids (VcGSTL3), with an average of 330.1 amino acids. The average molecular weight of blueberry GST proteins was 37.52 kDa, ranging from 18.06 kDa (VcGSTU16) to 98.99 kDa (VcGSTL3). The theoretical isoelectric points were from 4.66 (VcGSTU34) to 9.55 (VcDHAR). The aliphatic indices ranged from 71.83 (VcGSTL7) to 109.4 (VcGSTU52, VcGSTU57, VcGSTU72), and the grand average hydropathy values ranged from −0.519 (VcmPHES2_2) to 0.092 (VcGSTF11). A total of 81 GST proteins showed instability indices greater than 40, accounting for 45.51% of total proteins. The EF1BG subfamily had the largest average length (459.7 aa) and molecular weight (52.56 kDa) among the subfamilies. The TCHQD subfamily had the highest theoretical isoelectric point (8.98). The Theta subfamily showed the highest instability index (53.52) and aliphatic index (94.43), and the Phi subfamily showed the highest average grand hydropathy. Subcellular localization prediction suggested that most VcGST proteins were in a cytoplasmic location, followed by a chloroplastic location, with a few localized to the mitochondria, nucleus, and plasma membrane (Table S1).

### 2.2. Phylogenetic Analysis of GST Genes in Blueberry

A maximum likelihood phylogenetic tree was constructed using 178 blueberry GST proteins and 78 GST proteins from *Arabidopsis thaliana*, *Oryza Sativa*, *Brassica napus Solanum tuberosum*, and *S. lycopersicum* (Figure 1). Phylogenetic analysis showed that the GST family in blueberry could be divided into 12 subfamilies: DHAR, EF1BG, GHR, GST2N, Lambda, Metaxin, mPGES2, Phi, Tau, TCHQD, Theta, and Zeta. Among them, the Tau and Phi subfamilies were the two largest subfamilies, containing 74 genes (41.57%) and 22 genes (12.36%), respectively. The mPGES2 subfamily contained only 2 genes, which account for 1.12% of total *VcGSTs*. The member number in the rest subfamily ranged from 4 (TCHQD and Theta subfamily) to 16 (EF1BG subfamily). No hemerythrin and iota subfamily was found in the *VcGSTs*.

### 2.3. Chromosomal Localization, Gene Structure, and Conserved Motif Analysis of GST Genes

**Figure 2:**
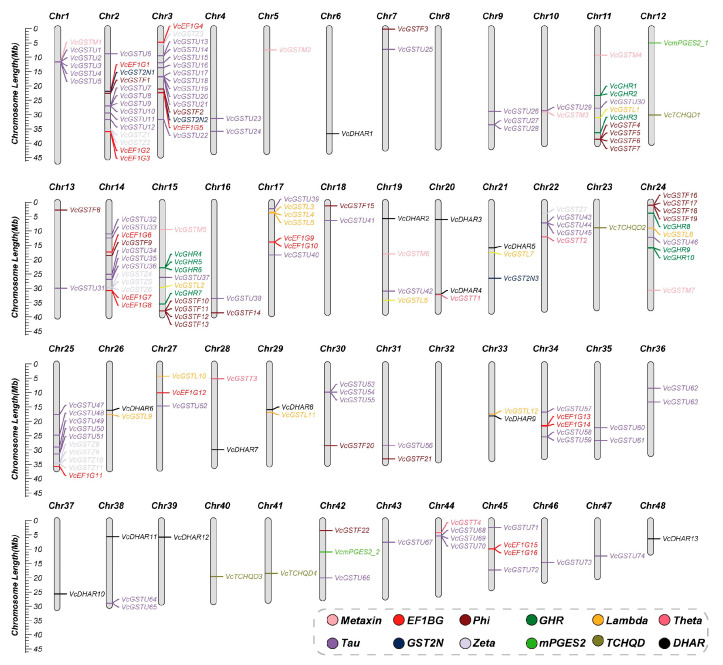
Chromosomal distribution of the 178 blueberry *VcGST* genes. Genes marked with the same color represent those that belong to the same subfamily.

Chromosomal localization analysis showed that *VcGSTs* were unevenly distributed on 46 chromosomes. No *VcGSTs* gene was found on chromosomes 8 and 32. The gene number of each chromosome ranged from 1 to 15 with an average of 3.87. Three chromosomes contained the highest number of *VcGST* genes and eleven chromosomes contained only one *VcGST* gene. Seven chromosomes included more than ten *VcGST* genes, accounting for 46.1% of the total number of *VcGST* genes (Figure 2). Subfamily classification showed that *VcGST* genes in the subfamily of Tau, Phi, DHAR, and Lambda were distributed most widely on 22 (45.8%), 13 (28.0%), 12 (25.0%), and 10 (20.8%) chromosomes, respectively. Moreover, we identified 13 *VcGST* gene clusters on 12 chromosomes, with each cluster’s genes being members of a single subfamily. Specifically, six clusters were comprised of Tau subfamily genes, three of Phi genes, and two of Zeta genes, while one exhibited GHR subfamily genes and one encompassed Lambda subfamily genes.

**Figure 3:**
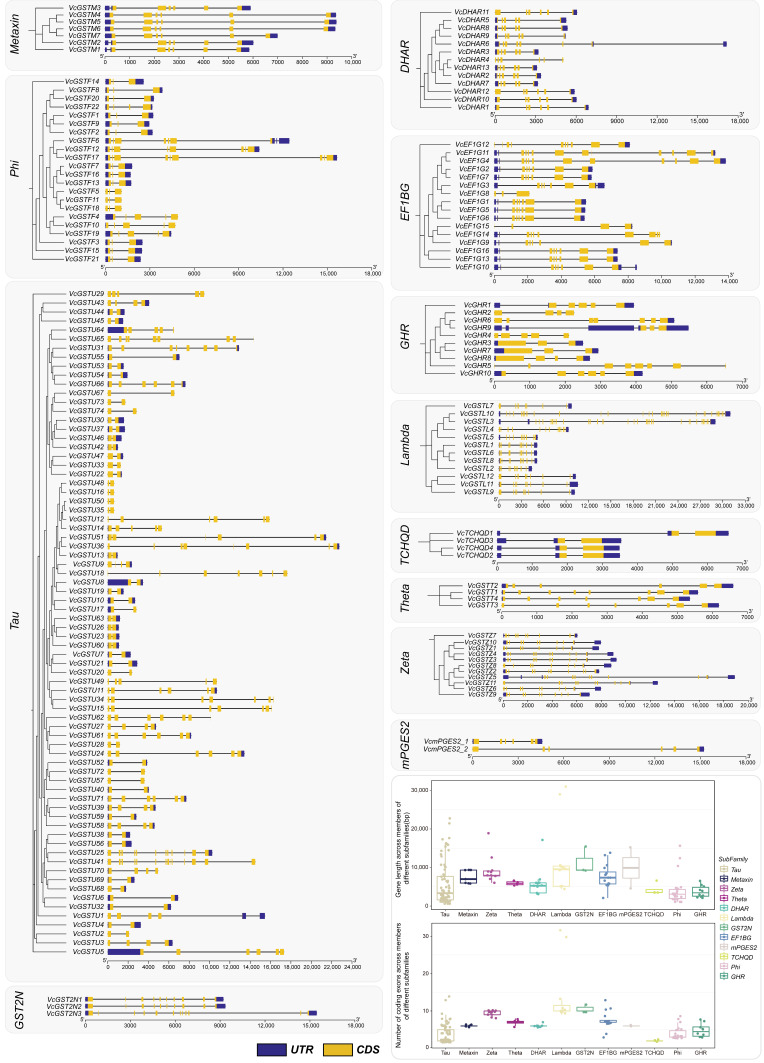
Gene structures, gene length, and exon numbers of the 178 *VcGST* genes. Genes from different subfamilies are separated using gray boxes. The box plots represent the gene length and number of coding exons among genes in different subfamilies. The subfamilies are distinguished by different x-axis labels and colors. Each point represents one *VcGST* gene, and its corresponding y-axis value indicates its gene length or exon number.

We also characterized the gene structures of *VcGSTs* (Figure 3). The results showed that the lengths of *VcGST* genes ranged from 606 bp (*VcGSTU48*, *VcGSTU50*, *VcGSTU35*, and *VcGSTU16*) to 31,011 bp (*VcGSTL10*), with an average length of 6387 bp. The *VcGST* genes in the subfamilies GST2N and Lambda were relatively longer than the genes in other subfamilies, with average lengths of 11,344 and 11,262 bp, respectively. By contrast, Phi and GHR subfamily members showed relatively shorter gene lengths, with average lengths of 4115 and 3771 bp, respectively. The exon numbers of *VcGST* genes ranged from 2 to 32, with 48 (27.0%) genes containing 2 exons. Two Lambda subfamily genes, *VcGSTL3* and *VcGSTL10,* contained markedly more exons than other *GST* genes, which have 32 and 30 exons, respectively. The *VcGST* genes in the subfamilies Lambda, GST2N and Zeta had higher exons numbers than the others, with average exon numbers of 13.8, 10.7, and 9.45, respectively. Genes in the subfamilies TCHQD, Tau, and Phi had relatively fewer exons, with average numbers of 2, 3.71, and 4, respectively. Additionally, exon numbers were conserved among DHAR, Metaxin, mPGES2, and TCHQD subfamily members, while exon numbers presented dramatic diversity in genes belonging to the subfamilies Phi, Tau, Lambda, GHR, and EF1BG. Additionally, untranslated regions were found in 150 *VcGST* genes, accounting for 84.3% of all *VcGSTs*.

**Figure 4:**
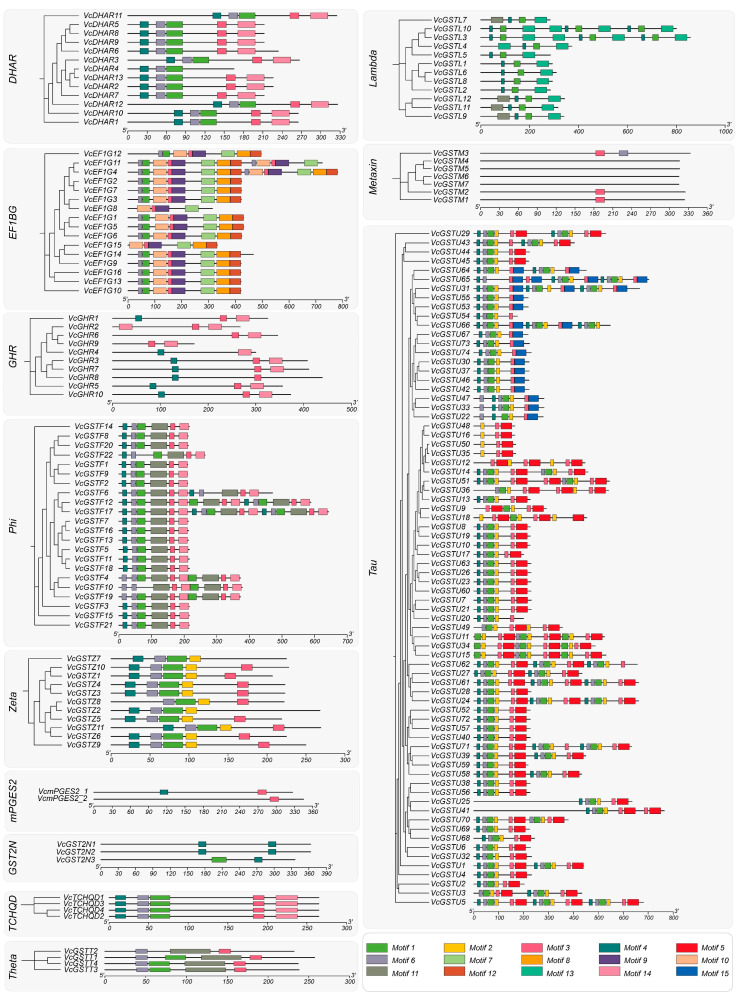
Conserved motifs distribution in the VcGST protein sequences. Different conserved motifs are marked using different-colored boxes.

We identified 15 conserved motifs in the VcGST protein sequences using the MEME (Figure 4, Table S2). The results showed that Motif 3, Motif 1, Motif 6, and Motif 4 were widely discovered in blueberry VcGST proteins. They were found in the 155 (87.1%), 150 (84.3%), 142 (79.8%) and 141 (79.2%) VcGST protein sequences, respectively. The distribution frequency ranged from 8.43% (Motif 13) to 51.1% (Motif 2) for the other motifs. Motif 4, Motif 1, Motif 3, and Motif 6 were ubiquitous across different subfamilies, as they were found in 11 (91.7%), 10 (83.3%), 10 (83.3%), and 8 (66.7%) subfamilies, respectively. In addition, Motif 15 was only found in the Tau subfamily, while Motifs 8, 9, and 12 were only found in the EF1BG subfamily. Different conserved motifs were widely distributed in the Tau, Phi, and EF1BG subfamilies, whereas only two motifs were distributed in the Metaxin subfamily, with no motifs found in VcGSTM4, VcGSTM5, VcGSTM6, or VcGSTM7.

**Figure 5:**
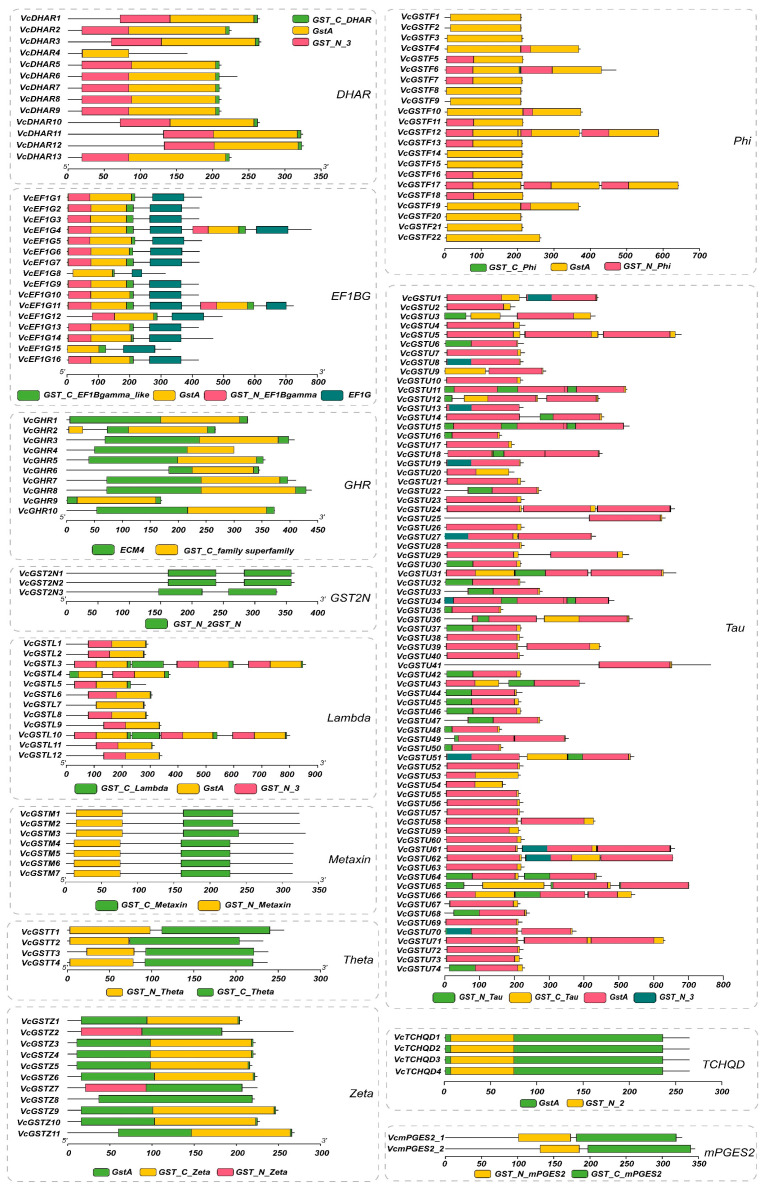
The conserved structural domains in the VcGST proteins from different subfamilies. Structural domains are marked with different-colored boxes.

The major conserved structural domains present in the blueberry GST proteins were also characterized. The results demonstrated that 178 of the identified VcGST proteins belonging to different subfamilies in this study possess specific GST-conserved structural domains. Combined with the analysis of conserved motifs, we found that motifs 1, 4, and 6 are primarily associated with the GST_N structural domain, while motifs 2, 3, 5, 9, 10, 11, 13, 14, and 15 are related to the GST_C structural domain. Notably, motifs 7, 8, and 12 are linked to the EF1G structural domain of the EF1BG subfamily (Figure 5).

### 2.4. Gene Function Enrichment Analysis

**Figure 6:**
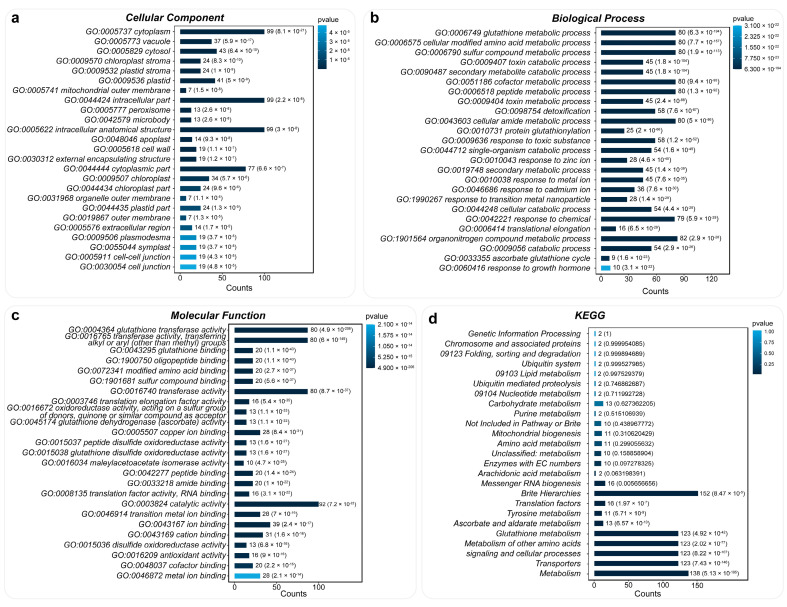
GO and KEGG enrichment analysis results for the 178 *VcGST* genes. Bar charts show the number of *VcGST* genes enriched in each term, with bar color representing the *p*-values. (**a**) shows term counts distribution in cellular component gene ontology; (**b**) shows term counts distribution in biological process gene ontology; (**c**) shows term counts distribution in molecular function gene ontology; (**d**) shows KEGG pathway enrichment analysis for *VcGST* genes.

A total of 178 blueberry *GST* genes were enriched in three categories: molecular function (MF), cellular component (CC), and biological process (BP). For the cellular component category, *VcGSTs* were mainly enriched in terms of the cytoplasm, intracellular part, intracellular anatomical structure, and cytoplasmic part (Figure 6a). Those four categories occupied 36.31% of all enriched items. In the biological process category, *VcGSTs* were extensively enriched in eight areas, including the glutathione metabolic process, cellular-modified amino acid metabolic process, and peptide metabolic process (Figure 6b). Four main terms were identified in the molecular function category, which encompassed glutathione transferase activity, transferase activity (GO:0016765 & GO:0016740), and catalytic activity (Figure 6c). Six types, which were mainly associated with amino acid metabolism and transport, were indicated in the KEGG enrichment analysis. These KEGG terms refer to glutathione metabolism, metabolism of other amino acids, signaling and cellular processes, transporters, and metabolism (Figure 6d).

### 2.5. Cis-Acting Element in the VcGST Promoter Regions

**Figure 7:**
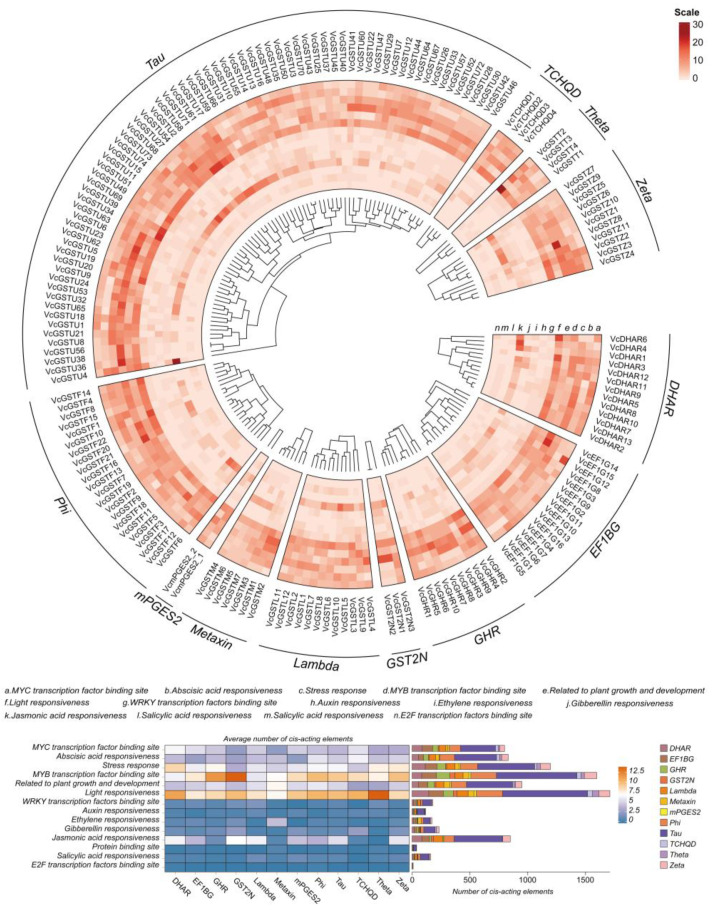
Cis-acting elements analyses of 2000 bp promoter regions of the 178 *GST* genes. All elements are classified into 14 types based on function. The circular heat map shows the distribution of the 14 types of elements in each gene. The rectangle heat map and stacked bar chart show the average number of different promoter types distributed in each subfamily and the total number distributed in different subfamilies.

Two thousand base-pairs promoter sequences upstream of the *VcGSTs* coding sequences were analyzed to gain deeper insights into gene transcriptional regulation. We discovered 25,708 cis-acting elements in the promoter regions of the 178 *VcGSTs*, among which 8863 functional elements were identified after the removal of common elements. The functional elements were clustered into 14 types, with most classed as hormone-responsive elements, transcription-factor binding sites, and abiotic response (Figure 7). Light responsiveness, MYB transcription-factor binding site, and stress response elements were the most abundant, accounting for 1713 (19.33%), 1600 (18.05%), and 1198 (13.52%) of all elements, respectively. Additionally, together with the elements mentioned above, MYC transcription-factor binding site and plant growth- and development-related elements were the most widely distributed of the five types. Subfamily analysis results for cis-acting elements indicated that light-responsiveness elements were distributed in 12 subfamilies. Members of the subfamilies GHR, GST2N, Phi, Tau, and Zeta contained more MYB transcription-factor binding site elements, while stress response elements were prevalent in the members of the DHAR subfamily.

### 2.6. Collinearity Analysis and Gene Duplication

**Figure 8:**
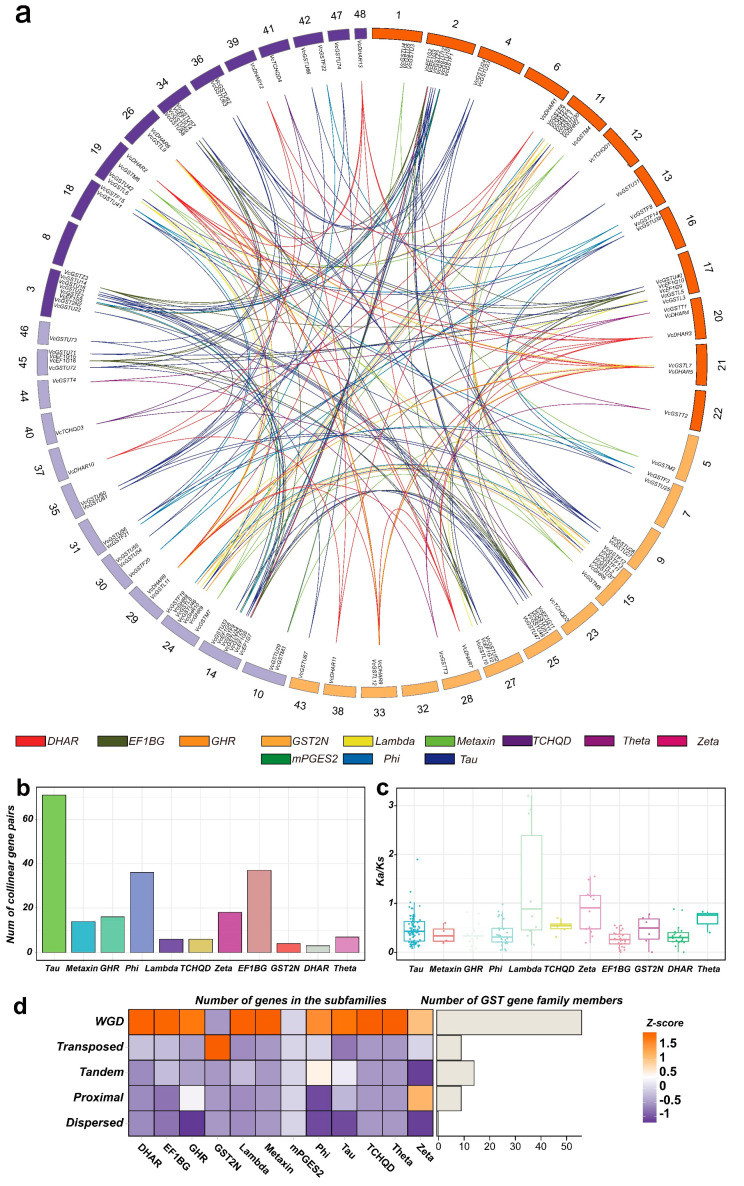
Evolution and duplication relationships of 178 blueberry *VcGST* genes. (**a**) shows paralogous gene pairs with collinear relationships, where pairs from different subfamilies are connected by lines in different colors; (**b**) shows the number of collinear gene pairs in different subfamilies; (**c**) shows the Ka/Ks values of paralogous pairs in boxplots, where pairs from different subfamilies are labeled in different colors, and each dot represents the Ka/Ks value of a gene pair; (**d**) shows the gene duplication types and numbers in different subfamilies in the heatmap.

Paralogous gene pairs within the *GST* family and the Ka/Ks values for those pairs were analyzed. A total of 218 paralogous gene pairs involving 131 *GST* genes were identified within the collinear blocks. Except for the mPGES and GST2N subfamilies, paralogous pairs were identified in all subfamilies, with the Tau, DHAR, and EF1BG subfamilies having relatively more pairs: 71, 36, and 37, respectively. Only three pairs were found in the GST2N subfamily (Figure 8a,b). The substitution rate calculation results showed that most *VcGST* gene pairs had Ka/Ks values less than one, indicating that these genes undergo purifying selection. Meanwhile, 14 gene pairs had Ka/Ks values greater than one, belonging to the Lambda, Zeta, and Tau subfamilies, suggesting these genes undergo positive selection (Figure 8c). Gene duplication analysis results revealed five different duplication types, among which whole-genome duplication (WGD) was the most prevalent event, with 56 *VcGST* genes identified as products of WGD. In addition, 9, 14, and 9 genes were identified to be derived from transposed duplication (TRD), tandem duplication (TD), and proximal duplication (PD). No genes generated by dispersed duplication (DSD) were identified in the blueberry *VcGST* genes. Additionally, no gene replication events of any of the five types were found in the mPGES2 subfamily. WGD is the dominant gene replication type in most subfamilies (Figure 8d).

**Figure 9:**
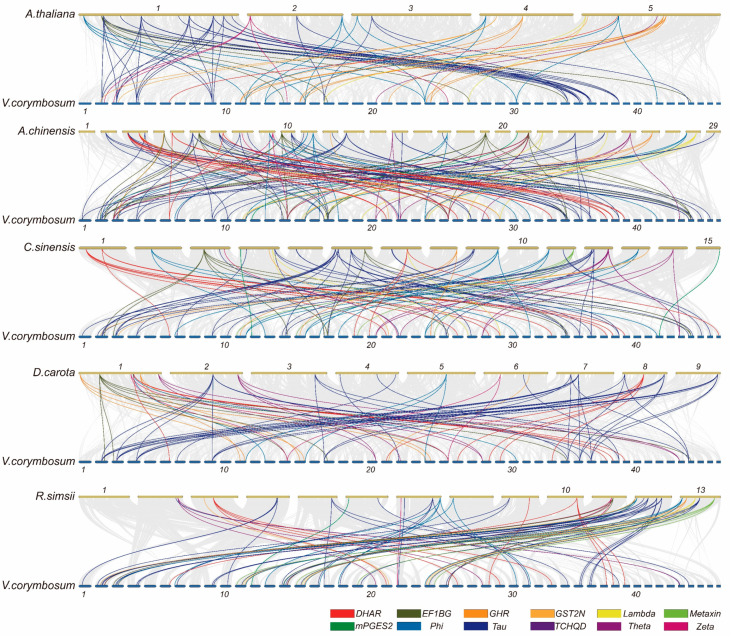
Collinearity analysis of *GST* genes between blueberry and *A. thaliana*, *A*. *chinensis*, *C*. *sinensis*, *D*. *carota* and *R*. *simsii*. The ribbons indicate collinear *GST* gene pairs between blueberry and other plant species. Collinear gene pairs belonging to different subfamilies are denoted with colored lines.

To further characterize the evolutionary relationships among *GST* genes across different species, we analyzed the *GST* genes collinearity between blueberry and *A. thaliana*, *Actinidia chinensis*, *Camellia sinensis*, *Daucus carota* and *Rhododendron simsii* (Figure 9). The results showed that 366, 142, 106, 79, and 92 collinear gene pairs were identified between paired species, respectively, involving 76, 83, 78, 49, and 89 blueberry *GST* genes. Seventeen genes exhibited collinearity relationships across all five species. Thirty-eight blueberry *GST* genes did not show collinearity with any other species, suggesting the evolution of these genes in blueberry may be species-specific (Table S3). Furthermore, the Tau subfamilies exhibited the most extensive collinear relationships across different species.

### 2.7. Expression Patterns of Blueberry VcGST Genes during Anthocyanin Accumulation and Potential GST Proteins for Anthocyanin Intracellular Transport

**Figure 10:**
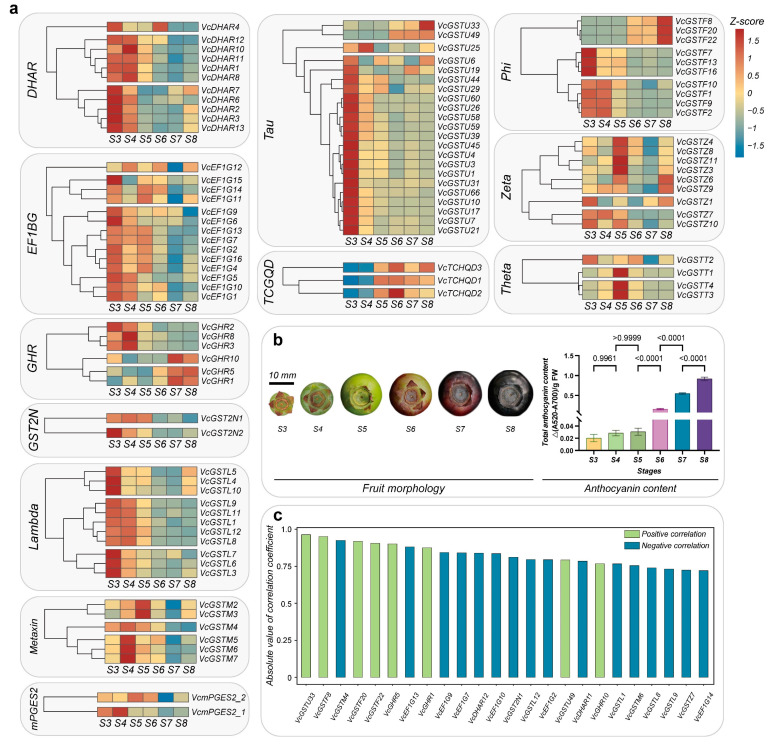
Expression patterns of 100 *GST* genes, anthocyanin content in ripening fruits, and their correlation analyses. (**a**) shows the expression patterns of different *VcGST* genes during fruit ripening, and the gene expression levels at different stages were internally normalized. The dendrogram on the left side of the heat map shows clustering results of the gene expression patterns; (**b**) shows morphological changes during fruit ripening and the anthocyanin content; (**c**) shows the correlation coefficients in bar charts, where light and dark green bars represent positive and negative correlations, respectively.

We characterized *VcGST* genes with relatively high expression levels at six stages of fruit development based on our previous transcriptomic data. Correlation coefficients between gene expression patterns and changes in anthocyanin content were also determined. One hundred *GST* genes with relatively high expression levels during fruit ripening were screened (Figure 10a). All subfamilies had members with high expression levels during blueberry fruit ripening. Genes in the families Phi and EF1BG had higher expression levels during fruit ripening, with total FPKM values of 1844 and 1291, respectively, followed by the subfamilies DHAR (786.8) and Tau (626.6). *VcGSTF8*, *VcGSTF2*, and *VcDHAR8* were the three genes with the highest expression levels, with total FPKM values of 689.7, 342.6, and 308.5, respectively. The genes in the DHAR, EF1BG, Lambda, Metaxin, and Tau subfamilies were mainly expressed at stages S3 or S4, while genes in the subfamilies Zeta and Theta were mainly expressed at stage S5. Genes in the subfamilies Phi and GHR showed dramatic intra-subfamily differences in their expression patterns. The expression levels of *VcGSTF8*, *VcGSTF20*, and *VcGSTF22* in the Phi subfamily gradually increased over time, while other genes were mainly expressed at stages S3 and/or S4. *VcGHR10*, *VcGHR5*, and *VcGHR1* in the GHR subfamily were mainly expressed at stages S7 and S8, while other genes were expressed at stages S3 and S4. The anthocyanin accumulated mainly from stage S6 to S8 and reached the highest level at stage S8 (Figure 10b). A total of 24 genes from 9 subfamilies with an absolute value of Pearson’s correlation coefficient greater than 0.7 between expression levels and anthocyanin content alterations were discovered. Of these, 8 genes were positively correlated with anthocyanin content changes, while 16 genes were negatively correlated with such changes (Figure 10c). Six genes that belonged to subfamilies Phi (*VcGSTF8*, *VcGSTF20* and *VcGSTF22*), Tau (*VcGSTU33*), Metaxin (*VcGSTM4*), and GHR(*VcGHR5*) with correlation coefficients greater than 0.9 were found.

**Figure 11:**
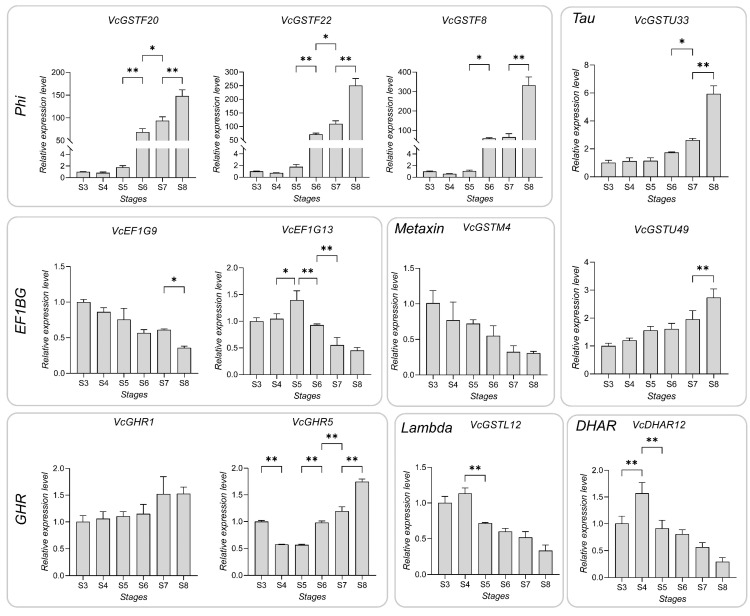
The qRT-PCR results of the 12 genes with high correlation between expression patterns and anthocyanin content from stages S3 to S8. Error bars indicate the standard deviation (S.D.) of three biological replicates. Significant differences in expression levels between two adjacent stages were tested using one-way ANOVA. The asterisk and the double asterisks represented significant differences under *p* < 0.05 and *p* < 0.01, respectively.

We also performed quantitative real-time PCR (qPCR) analysis on 12 genes with expression patterns highly correlated with anthocyanin content. The qPCR results were significantly consistent with the transcriptomic expression profile. Specifically, *VcGSTF20*, *VcGSTF22* and *VcGSTF8* in the subfamily Phi, *VcGSTU33*, and *VcGSTU49* in the Tau subfamily, and *VcGHR1* in the GHR subfamily, were upregulated during fruit ripening stages, peaking at S8 and positively correlating with anthocyanin accumulation. *VcGHR5* was generally upregulated following fruit ripening, with the exception of stage S3, and probably showed a positive correlation with the anthocyanin amount (Figure 11, Table S4). The expression level of *VcGSTF20*, *VcGSTF22*, and *VcGSTF8* at stages S3, S4 and S5 were low while sharply increased from S6 to S8. *VcGSTF20*, *VcGSTF22,* and *VcGSTF8* were upregulated by 148-, 250-, and 366-fold at stage S8 compared to S3. The expression levels of *VcEF1G13*, *VcGSTM4*, *VcGSTL12*, and *VcDHAR12* peaked at stage S5 or S4 and showed downward trends thereafter.

**Figure 12:**
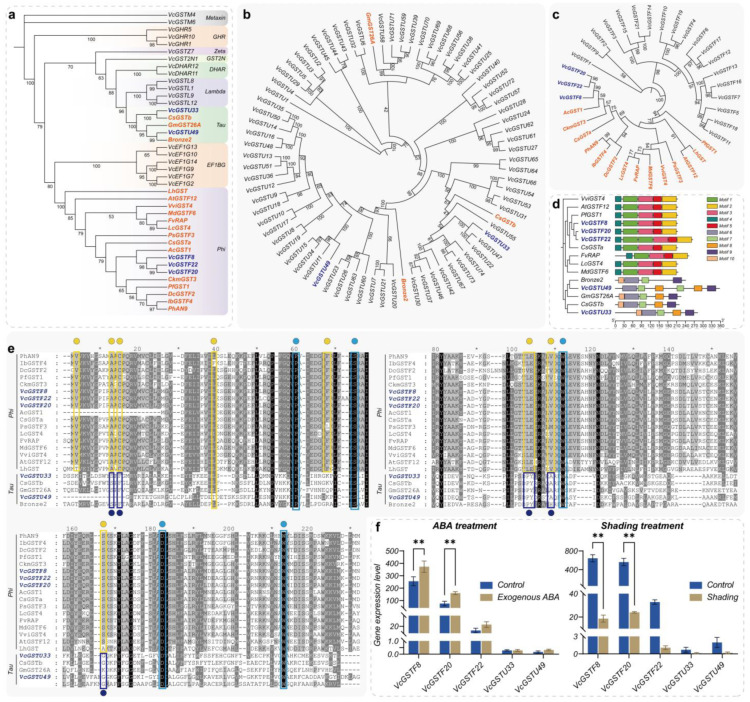
Potential anthocyanin intracellular transport *VcGST* genes in blueberries. (**a**) shows the phylogenetic tree of 24 VcGSTs that are correlated with anthocyanin content and 17 validated anthocyanin intracellular transport GST proteins. Validated anthocyanin intracellular transport GST proteins were marked in orange, and blueberry VcGSTs clustered in the same clade were marked in purple. The digits on the branches represent bootstrap values based on 1000 iterations; (**b**,**c**) show the phylogenetic tree of validated anthocyanin intracellular transport GSTs and blueberry VcGSTs in the Phi and Tau subfamilies; (**d**) shows the conserved motifs of the 15 anthocyanin intracellular transport GSTs; (**e**) displays the conserved residues of GST proteins, with those proven to be associated with anthocyanin intracellular transport highlighted with yellow dots and boxes. The conserved residues of GSTs belonging to the Tau subfamily are marked with purple dots and boxes. The conserved residues common to GST proteins are indicated by blue dots and boxes; (**f**) shows the expression levels of five potential anthocyanin intracellular transport *GSTs* in mature blueberry fruits under exogenous ABA and whole-plant shading treatments. Error bars indicate the standard deviation (S.D.) of the mean of three transcriptome sample data. The significance of differential expression between two adjacent stages was calculated using two-way ANOVA. The asterisk and the double asterisks represented significant difference under *p* < 0.05 and *p* < 0.01, respectively.

We selected 17 GST proteins that were validated to be involved in anthocyanin intracellular transport and constructed a phylogenetic tree with the 24 VcGSTs (Figure 12a). The results revealed that previously reported anthocyanin intracellular transport GST proteins belong to the Phi and Tau subfamilies. VcGSTF8, VcGSTF22, and VcGSTF20 were clustered with 14 Phi proteins. VcGSTU49 was clustered with Bronze2 and GmGST26A and VcGSTU33 was clustered with CsGSTb. To figure out the influence of phylogenetic closeness within subclasses on clustering patterns, we performed phylogenetic analysis using all Phi and Tau subclass members in the blueberry GST family, together with respective anthocyanin intracellular transporters. VcGSTU33 and CsGSTb, VcGSTU49, and Bronze2 were clustered separately from each other with relatively long phylogenetic distances (Figure 12b). The results showed VcGSTF8, VcGSTF22, and VcGSTF20 were closely related to 14 anthocyanin intracellular transporter Phi proteins, while they were distinct from the other 19 blueberry Phi members (Figure 12c). Conserved motifs of anthocyanin intracellular transporters were analyzed (Figure 12d). VcGSTF8, VcGSTF20, and VcGSTF22 share highly similar motif compositions with seven other Phi proteins. VcGSTF22 contains two repeated instances of Motif 1, while VcGSTU49 showed distinct patterns. VcGSTU33 displayed similar motif profiles to three other Tau transporters. We also compared those 5 VcGSTs proteins to 17 known anthocyanin transporters (Figure 12e). The results showed that all proteins share conserved residues of GST proteins (blue boxes). VcGSTF8, VcGSTF22, and VcGSTF20 possess conserved residues identified in known anthocyanin intracellular transporters (yellow boxes). GST proteins in the Tau subfamily largely do not share the conserved residues of Phi members in the conserved sites shown here (purple boxes). The expression levels of anthocyanin intracellular transport protein genes are closely related to the anthocyanin content. Studies have shown that exogenous ABA and whole-plant shading, respectively, significantly increase and decrease the anthocyanin content in blueberries. To further predict blueberry anthocyanin intracellular transport GST proteins, we characterized the expression levels of *VcGSTF8*, *VcGSTF20*, *VcGSTF22*, *VcGSTU33*, and *VcGSTU49* in blueberry fruit under exogenous ABA and whole-plant shading conditions, based on existing transcriptome data (Figure 12f). The results indicated that under ABA and shading conditions, the expression levels of *VcGSTF8*, *VcGSTF20*, and *VcGSTF22* were significantly upregulated and downregulated, respectively, in line with the changes in anthocyanin content. Notably, *VcGSTF8* and *VcGSTF20* showed high expression levels under different conditions, with very significant upregulation/downregulation. By contrast, *VcGSTU33* and *VcGSTU49* displayed low expression levels under both conditions, with no significant changes in response to ABA or shading.

## 3. Discussion

### 3.1. Identification and Characterization of the Blueberry VcGST Genes

The glutathione S-transferase (*GST*) gene family is a complex and diverse group of genes that has been proven to participate in regulating plant growth and development, abiotic stress tolerance, secondary metabolism, and signal transduction. To date, the *GST* gene family has been identified and characterized in several plant species, including Arabidopsis [29], rice [30], strawberries [31], and cucumbers [32]. However, prior to our study, the *GST* gene family in blueberry had yet to be comprehensively identified and analyzed. In this study, we identified 178 *GST* genes from the blueberry genome and classified them into 12 classes based on phylogenetic relationships. The Tau and Phi subfamilies were known as plant-specific GSTs, containing the largest number of members followed by EF1BG, DHAR, and Lambda. Similar results were also reported for other plant species. The roles of Tau and Phi subfamily members have been extensively studied. The Phi subfamily member *PpGST1* and the Tau subfamily member *PpGST2* are involved in sugar response, salicylic acid (SA), and indole-3-acetic acid (IAA) signaling during pear fruit development [33]. Two Tau subfamily GST proteins participate in the cellular transport of trans-Resveratrol in grape [34]. Additionally, GST proteins predominantly categorized within these two subfamilies were reported to be associated with anthocyanin intracellular transport functions in various fruit species, including litchi [23], kiwifruit [35], peaches [36], and strawberries [37]. Those studies indicated that Tau and Phi probably play unique and important roles in the plant kingdom. Phylogenetic similarity and protein motifs conservation among genes in the same subfamily indicated a common ancestor and possibly similar functions in plants. Cis-acting elements analyses of the *VcGST* promoters indicated the gene expression was probably induced by light and phytohormone signals. MYB and MYC transcription-factor binding sites were highly enriched in the blueberry GST gene promoters, implying that they potentially play significant roles in regulating *VcGST* gene expression. Aside from light-responsive and MYB binding sites, stress-responsive elements comprised the most abundant category and were widely distributed in the subfamilies DHAR, Theta, and Zeta, suggesting possible roles for these three subfamilies in stress response; similar findings were also reported in melon [38], cucumber [32], and tomato [39], indicating conserved roles for GSTs among fruit trees.

Changes in gene structure are mainly caused by three mechanisms: insertion/deletion, exon/pseudogene, and exon/intron gain/loss, with the number of mutational events for each gene largely being proportional to evolutionary time. Genes with more complex structures may have arisen more recently in evolution compared to those with simpler structures [40]. Additionally, divergence in gene structure can generate proteins with distinct sequence features, playing an important role in the diversification of multi-gene families [41]. The exon numbers were relatively conserved within some subfamilies containing DHAR, GST2N, Metaxin, mPGES2, TCHQD, Theta, and Zeta. However, the exon numbers varied markedly within the EF1BG, GHR, Lambda, Phi, and Tau subfamilies. Genes in the Tau subfamily possessed 2 to 14 exons. The results showed noticeable differences in the number of *GST* genes among subclasses, with the Lambda subfamily having the highest mean number of exons, while Phi, Tau, and TCHQD had relatively fewer average exons. This suggested that *VcGST* members in the subfamilies Phi, Tau, and TCHQD possibly experienced more evolutionary events.

Previous studies have shown that gene duplication events are the primary cause of gene family expansion. Different types of gene duplication (*i.e.*, TD, PD, DSD, WGD, and TRD) play vital roles in the expansion of plant gene families [42,43]. Additionally, whole-genome duplication, segmental duplication, and tandem duplication have been identified as the major mechanisms of gene duplication during gene family expansion [44]. WGD was the predominant mechanism for most subfamilies, although no duplications were identified in the subfamily mPGES2 and mainly TRD events were observed in the subfamily GST2N. Furthermore, we identified 218 duplicate gene pairs located in syntenic blocks among the *GST* family. Synteny refers to homologous genes that are located in corresponding chromosomal regions and have similar relative positioning between species or within a species. Duplicate genes in syntenic blocks were likely derived from WGD or segmental duplications [45]. The 218 syntenic duplicate pairs involved 131 non-redundant *GST* genes. Together with the 56 *GST* genes generated through previously identified WGD events, we speculate that segmental duplication also contributed significantly to the expansion of blueberry *GST* genes. In summary, WGD and segmental duplication appear to be the primary mechanisms driving the expansion of the *GST* gene family in blueberries.

### 3.2. Potential Anthocyanin Intracellular Transporters in the Blueberry GST Gene Family

Anthocyanins are water-soluble pigments that confer vibrant colors to plant leaves, flowers, and fruits, serving as visual signals that attract pollinators and enable the dispersal of seeds [46,47]. In addition to indispensable biosynthetic processes, the transport of anthocyanins from the endoplasmic reticulum where they are synthesized to the acidic vacuoles is essential to prevent oxidation and achieve their function as pigments [48]. While anthocyanin biosynthesis pathways have been extensively characterized and proven to be conserved in plants, the intracellular transport mechanisms of anthocyanins remain elusive. In recent years, three potential pathways, including glutathione S-transferase mediated transport, vesicular trafficking, and membrane transporter-mediated transport, were found to be involved in anthocyanin intracellular transport in some plants [20]. Among them, the role of GST proteins in anthocyanin intracellular transport has been relatively well studied, with transporter genes identified in many species like maize [49], Arabidopsis, litchi, grape, kiwifruit, apple, etc., suggesting the GST-mediated pathway is likely conserved in plants. The expression levels of anthocyanin intracellular transporter genes are closely correlated with the anthocyanin content. In summary, we predicted that VcGSTF8, VcGSTF20, and VcGSTF22 might be anthocyanin intracellular transport proteins in blueberries. Our study would lay a foundation for further study on VcGSTs’ function and the mechanism of anthocyanin intracellular transport in blueberries. However, their roles in blueberry anthocyanin intracellular transport still need further functional verification.

## 4. Materials and Methods

### 4.1. Identification and Characterization of GST Genes in Blueberry

Amino acid sequences of blueberry (*V*. *corymbosum cv.* ‘Draper’) were downloaded from the Vaccinium genome database (https://www.vaccinium.org, accessed on 5 May 2023). A local blueberry amino acid database was constructed using the makeblastdb command in the BLAST+ 2.13.0 toolkit. Sixty-two GST protein sequences of *A. thaliana* from The Arabidopsis Information Resource (TAIR, https://www.arabidopsis.org/, accessed on 5 May 2023), nine OsGST protein sequences from the Rice Genome Annotation Project Database (RGAP, http://rice.uga.edu/, accessed on 5 May 2023), and seven BnGST protein sequences identified by Wei et al. [6] were used as queries to find homologous blueberry proteins in the local database with an E-value cutoff of 1 × 10^−10^ and an identify cutoff of more than 50%. Besides, hidden Markov model (HMM) profiles for the GST-N domain (PF02798) and GST-C domain (PF00043) were obtained from the InterPro database (https://www.ebi.ac.uk/interpro/, accessed on 5 May 2023) and used for search sequences with HMMER 3.3.2 [50] at an E-value cutoff of 1 × 10^−10^. After manually removing duplicated and incomplete sequences, all candidate blueberry GST proteins were uploaded to Batch CD-Search Tool (https://www.ncbi.nlm.nih.gov/Structure/bwrpsb/bwrpsb.cgi, accessed on 15 May 2023) for domain validation. The physicochemical properties of the blueberry GST proteins were calculated using ExPasy (https://www.expasy.org/, accessed on 18 May 2023). Subcellular localizations of the GST proteins were predicted using WoLF PSORT (https://wolfpsort.hgc.jp/, accessed on 19 May 2023).

### 4.2. Multiple Sequence Alignment, Phylogenetic Analysis, and Subfamily Classification

A total of 256 GST amino acid sequences from *A. thaliana*, *O*. *sativa*, *B*. *napus*, *S*. *tuberosum* [51], *S. lycopersicum* [39], and *V. corymbosum* were aligned using MAFFT 7.520 [52]. A phylogenetic tree with 1000 bootstrap replicates was constructed using IQ-TREE.2.3.2 [53] under an amino acid substitution model VT+R4. Blueberry GST proteins were classified into subfamilies according to the known GST classification of *A. thaliana*, *O. sativa*, and *B. napus*. The phylogenetic tree was visualized and complemented using iTOL (https://itol.embl.de/, accessed on 2 June 2023).

### 4.3. Chromosomal Localization, Gene Structure, and Conserved Motif Analysis

The genome annotation file of the blueberry was downloaded from the Vaccinium genome database to extract chromosomal localization and gene feature information for the 178 *VcGSTs*. MapChart 2.32 [54] was used to plot the gene chromosomal localization. Gene structures were visualized using the online Gene Structure Display Server 2.0 [55] (GSDS, http://gsds.gao-lab.org/, accessed on 12 June 2023). Conserved motifs of VcGST proteins were characterized using the online tool MEME [56] (https://meme-suite.org/meme/tools/meme, accessed on 20 June 2023) with the maximum number of motifs setting as 15, while the motif lengths ranged from 6 to 50 amino acids. Other parameters were kept as default. TBtools [57] was used to plot the conserved motifs among GST proteins.

### 4.4. Gene Function Annotation, Cis-Regulatory Element Analysis, and Duplication Event Analysis

The blueberry GST genes were functionally annotated using Gene Ontology (GO) and Kyoto Encyclopedia of Genes and Genomes (KEGG) pathway. GO annotations were obtained from the EGGNOG-MAPPER server (http://eggnog-mapper.embl.de/, accessed on 29 June 2023) using an E-value cutoff of 1 × 10^−5^. KEGG pathways were assigned to GST genes using the KEGG Automatic Annotation Server (KAAS, https://www.genome.jp/tools/kaas/, accessed on 3 July 2023). Enrichment analysis of GO terms and KEGG pathways was performed and the results were visualized using the R package clusterProfile [58]. For cis-element analysis, 2000 bp promoter sequences upstream of the *VcGST* coding regions were extracted using TBtools and analyzed using the online tool PlantCARE (http://bioinformatics.psb.ugent.be/webtools/plantcare/html/, accessed on 15 July 2023). Data visualization was performed with the R package circlize [59]. MCSCANX [45] was used to analyze the collinearity and duplication events within the ‘Draper’ blueberry genome and between other species’ genomes. The *A. thaliana* genome data used in the study were downloaded from the Ensembl Plants database (https://plants.ensembl.org/index.html, accessed on 27 July 2023), with the version number TAIR10. The *A. chinensis* genome data were downloaded from the Kiwifruit Genome Database (https://kiwifruitgenome.org/, accessed on 27 July 2023), with the version number Hong Yang v2. The *C. sinensis* genome data were obtained from the Plant GARDEN database (https://plantgarden.jp/, accessed on 27 July 2023), with the version number CSS_ChrLev_20200506. The *D. carota* genome data were downloaded from the National Center for Biotechnology Information database (NCBI, https://www.ncbi.nlm.nih.gov/, accessed on 27 July 2023), with the version number ASM162521v1. The *R. simsii* genome data were acquired from the Rhododendron Plant Genome Database (RPGD, http://bioinfor.kib.ac.cn/RPGD/download_genome.html, accessed on 27 July 2023). Duplication types were identified using DupGen_finder [60]. The ratio of non-synonymous substitutions per non-synonymous site (Ka) to synonymous substitutions per synonymous site (Ks) (Ka/Ks) of duplicated gene pairs was calculated using KaKs_Calculator 3.0 [61]. Circos 0.69–9 [62] was used to draw and visualize the results.

### 4.5. Plant Materials

Southern highbush blueberry (*V. corymbosum cv.* ‘Star’) fruits were sampled from 6-year-old blueberry plants grown in the blueberry germplasm repository at Zhejiang Normal University (29°10′ N, 119°44′ E). The plants were cultivated under standard cultural practices with an average temperature of 22–28 °C during the growing season. According to the fruit developmental stages defined by Zifkin et al. [63], fruits from stage three (S3, green fruit) to eight (S8, mature fruit) were randomly collected from different parts of the plants between 8:00 and 10:00 A.M. from late April to the middle of May in 2023. For each stage, 100 fruits were collected from 10 plants. The fruits were immediately cut into small pieces, wrapped in aluminum foil, and frozen in liquid nitrogen. Blueberry fruits were brought to the lab and stored at −80 °C until further use.

### 4.6. Anthocyanin Content Determination in Blueberry Fruits

To determine the anthocyanin content during blueberry fruit ripening, 35 fruits of uniform size were selected at each of the six developmental stages (S3–S8) with three biological replicates. For each replicate, 0.15 g of blueberry fruit powder was obtained by pooling and grinding an equal amount of fruits from the same stage. The fruit powder was placed in 1.5 mL of 99:1 (volume–volume) methanol–hydrochloric acid solution. The mixture was stored at 4 °C in darkness for 24 h then centrifuged at 4 °C and 9000 rpm for 10 min. Then, 200 μL of the supernatant was separately added to 800 μL of pH 1.0 KCl buffer and 800 μL of pH 4.5 sodium acetate buffer. Following the method of An et al. [64], Multiskan™ GO (Thermo Fisher Scientific, Waltham, MA, USA) was used to measure the anthocyanin content at wavelengths of 520 nm and 700 nm with six replicates for each stage. The total anthocyanin content was calculated according to the following equation:

Total anthocyanin content = [(A520 − A700)_pH 1.0_ − (A520 − A700)_pH 4.5_] (1)

### 4.7. RNA-Seq Data Analysis and Pearson Correlation Analysis

The raw RNA-seq data obtained during fruit ripening were sequenced in our laboratory. Raw sequencing reads were filtered and processed for quality control using Fastp 0.23.2 [65]. The reference genome of ‘Draper’ was indexed using Bowtie2 1.3.1 [66] and sequencing reads were aligned to the reference genome. Transcriptome quantification was performed using the featureCounts program in the subread package 2.0.3 [67]. The quantification data were normalized to Fragments Per Kilobase of exon model per Million mapped fragments (FPKM). Based on the normalized transcriptome data, expression data for the *VcGSTs* were extracted. *VcGSTs* with total FPKM values greater than five across the six developmental stages were selected for correlation analysis with fruit anthocyanin content. A heat map of gene expression was generated using the R package pheatmap. Correlation analysis between *VcGST* expression and blueberry anthocyanin content was performed using customized Python scripts.

### 4.8. RNA Extraction, cDNA Synthesis, and Real-Time Quantitative PCR Analysis

The total RNA was extracted from blueberry fruits at six stages using polysaccharide polyphenol total RNA extraction kits (TIANGEN, Beijing, China). First-strand cDNA was synthesized using cDNA synthesis kits (Vazyme, Nanjing, China). Twelve *VcGST* genes whose expression was highly correlated with fruit anthocyanin content were selected. Primers (Table S5) used for qRT-PCR were designed using the online tool Primer-BLAST and synthesized at Sangon Biotech (Shanghai, China). Primer specificity was verified by common PCR and qRT-PCR melting curves. Gene quantification was performed on a QuantStudio^TM^ 1 Real-Time PCR System. The reaction volume was 10 μL, including 5 μL SYBR Green qRT-PCR Premix, 0.2 μL forward and reverse primers, 1 μL cDNA template, and 3.6 μL double-distilled water. *VcGAPDH* was used as the internal reference gene [68]. The reaction procedure was 95 °C for 10 min, followed by 40 cycles of 95 °C for 30 s, 59 °C for 30 s, and 72 °C for 30 s. Four biological replicates were set for cDNA samples at each developmental stage. Relative gene expression was calculated using the 2^−ΔΔCt^ method. Data analysis and visualization were performed using R packages.

### 4.9. Potential Anthocyanin Intracellular Transport GST Proteins Identification and Analysis in Blueberry

MAFFT 7.520 was used to align the amino acid sequences of 17 GST proteins confirmed to be involved in anthocyanin intracellular transport (Table S6) and 24 VcGST proteins highly correlated with changes in anthocyanin content. A phylogenetic tree was constructed using 1000 bootstrap replicates in IQ-TREE 2.3.2, applying the amino acid substitution model Q.pfam+G4. To elucidate the impact of phylogenetic closeness within subfamilies on clustering patterns, phylogenetic analyses were conducted for all blueberry Phi and Tau subfamily members and their respective anthocyanin intracellular transport proteins using the same approach, with the best amino acid substitution models being LG+G4 and JTT+F+I+G4, respectively. The online tool MEME (accessed on 25 January 2024) was employed to identify conserved motifs in the amino acid sequences, setting the maximum number of motifs to 10 with lengths ranging from 6 to 50 amino acids. GeneDoc 2.7.0 was used to visualize the amino acid sequence alignments and the conserved residues were manually annotated. Exogenous ABA treatment of fruit [69] and whole-plant shading [70] have been reported to affect anthocyanin accumulation in blueberry fruits. Raw RNA-Seq data from validated exogenous ABA treatment were downloaded from the National Center for Biotechnology Information BioProject database under accession numbers PRJNA664011. The reads were processed using the method described in Section 4.7 to obtain normalized expression matrices; transcriptome data of fruit under shading treatment were obtained from the Supplementary Information of the original article. An R script was utilized to extract expression data for five potential blueberry anthocyanin intracellular transport *GST* genes (*VcGSTF8*, *VcGSTF20*, *VcGSTF22*, *VcGSTU9*, and *VcGSTU49*) and to conduct data analyses.

## 5. Conclusions

In this study, we identified 178 glutathione S-transferase (*GST*) genes within the ‘Draper’ blueberry genome, revealing an uneven chromosomal distribution across 46 chromosomes. Tau and Phi were the two largest subfamilies within the 12 identified subfamilies. *VcGSTs* were enriched mainly in glutathione metabolic process, peptide metabolic processes, and transferase activities. Cis-acting element analyses suggested that *VcGSTs* were primarily regulated by MYB and MYC transcription factors and were sensitive to light and/or stresses. *VcGSTs* were mainly expanded by WGD or segmental duplication events and most *VcGSTs* evolved under purifying selection. Combining the expression patterns of VcGSTs and their correlation with changes in anthocyanin content, along with further evolutionary and sequence analysis, we speculate that VcGSTF8, VcGSTF20, and VcGSTF22 are potential anthocyanin intracellular transport GST proteins in blueberries. This research lays a solid groundwork for advancing the understanding of anthocyanin intracellular transport mechanisms and exploring the potential functions of VcGSTs in blueberries.

## Data Availability

The RNA-seq dataset of blueberry fruit ripening generated in this study has been deposited in the National Genomics Data Center (NGDC) database and is accessible through the GSA accession number CRA010224 (https://bigd.big.ac.cn/gsa/browse/CRA010224, accessed on 22 March 2023).

## Supplementary Materials

The following supporting information can be downloaded at https://www.mdpi.com/article/10.3390/plants13101316/s1, Table S1: Analysis of protein physicochemical properties and prediction of subcellular localization of VcGSTs; Table S2: Fifteen conserved motifs of VcGST proteins; Table S3: Interspecies collinearity analysis; Table S4: Pearson correlation coefficients between relative expression changes of 12 genes and anthocyanin accumulation; Table S5: Gene primers used for real-time fluorescence quantification; Table S6: The 17 identified anthocyanin intracellular transport GST proteins used for the analysis of potential anthocyanin transport GST proteins in blueberries. References [71,72,73,74,75,76,77,78,79,80,81,82] are cited in the supplementary materials.
